# Short-course rapamycin treatment enables engraftment of immunogenic gene-engineered bone marrow under low-dose irradiation to permit long-term immunological tolerance

**DOI:** 10.1186/s13287-017-0508-3

**Published:** 2017-03-09

**Authors:** Kunal H. Bhatt, Rajeev Rudraraju, Jeremy F. Brooks, Ji-Won Jung, Ryan Galea, James W. Wells, Raymond J. Steptoe

**Affiliations:** The University of Queensland Diamantina Institute, The University of Queensland, Translational Research Institute, Brisbane, QLD Australia

**Keywords:** Bone marrow transplant, Gene therapy, Immune tolerance

## Abstract

**Background:**

Application of genetically modified hematopoietic stem cells is increasingly mooted as a clinically relevant approach to protein replacement therapy, immune tolerance induction or conditions where both outcomes may be helpful. Hematopoietic stem and progenitor cell (HSPC)-mediated gene therapy often requires highly toxic pretransfer recipient conditioning to provide a ‘niche’ so that transferred HSPCs can engraft effectively and to prevent immune rejection of neoantigen-expressing engineered HSPCs. For widespread clinical application, reducing conditioning toxicity is an important requirement, but reduced conditioning can render neoantigen-expressing bone marrow (BM) and HSC susceptible to immune rejection if immunity is retained.

**Methods:**

BM or HSPC-expressing OVA ubiquitously (actin.OVA) or targeted to MHC II+ cells was transferred using low-dose (300 cGy) total body irradiation. Recipients were administered rapamycin, cyclosporine or vehicle for 3 weeks commencing at BM transfer. Engraftment was determined using CD45 congenic donors and recipients. Induction of T-cell tolerance was tested by immunising recipients and analysing in-vivo cytotoxic T-lymphocyte (CTL) activity. The effect of rapamycin on transient effector function during tolerance induction was tested using an established model of tolerance induction where antigen is targeted to dendritic cells.

**Results:**

Immune rejection of neoantigen-expressing BM and HSPCs after low-dose irradiation was prevented by a short course of rapamycin, but not cyclosporine, treatment. Whereas transient T-cell tolerance developed in recipients of OVA-expressing BM administered vehicle, only when engraftment of neoantigen-expressing BM was facilitated with rapamycin treatment did stable, long-lasting T-cell tolerance develop. Rapamycin inhibited transient effector function development during tolerance induction and inhibited development of CTL activity in recipients of OVA-expressing BM.

**Conclusions:**

Rapamycin acts to suppress acquisition of transient T-cell effector function during peripheral tolerance induction elicited by HSPC-encoded antigen. By facilitating engraftment, short-course rapamycin permits development of long-term stable T-cell tolerance.

**Electronic supplementary material:**

The online version of this article (doi:10.1186/s13287-017-0508-3) contains supplementary material, which is available to authorized users.

## Background

Gene therapy approaches employing genetically modified hematopoietic stem and progenitor cells (HSPCs) show great promise for expression of therapeutic proteins within the hematopoietic system. Notable clinical successes have been achieved with therapy of severe combined immunodeficiency (scid)-X1 [1], leukodystrophies [2, 3] and Wiskott–Aldrich syndrome [4]. Potential applications tested in preclinical models are more diverse, encompassing a range of blood disorders including hemophilia and sickle-cell disease [5] and immunological disorders [6].

Enforced expression of antigen either ubiquitously or targeted to antigen-presenting cells (APC) of the immune system is a robust approach to inducing immune tolerance which prevents priming of T-cell and B-cell responses to expressed proteins [7, 8]. This has the power to prevent development of autoimmune disease in elicited and spontaneous models leading to proposals for therapeutic application [9, 10]. Indeed, under certain conditions established memory T-cell responses can be turned off [11], suggesting that enforced antigen expression may provide unique opportunities to control otherwise difficult-to-treat memory T-cell responses. For therapeutic application, an efficient means to achieve de-novo enforced antigen expression is HSPC-based gene therapy [12].

A critical requirement for clinical application of HSPC-based gene therapies, particularly if intended for therapy of nonlife-threatening diseases, is to minimise the toxicity of procedures associated with HSPC transfer. Currently for HSPC-mediated gene therapies, highly toxic pretransplant conditioning that both myeloablates and immunoablates patients prior to HSPC transfer is typically used [3, 4]. This facilitates high levels of engraftment of transferred HSPCs along with substantial replacement of recipient hematopoietic cells with those derived from the transferred, engineered HSPCs respectively. In some disorders, such as those where substantial replacement of long-lived hematopoietically derived cells might be required, immunoablation may be advantageous [4, 13], but may not be required in, for example, scid, where progeny of engineered HSPCs have a competitive advantage and could more easily repopulate recipients [1]. In other disorders where large-scale replacement of immune cells is not required, immunoablation is disadvantageous because it would be preferable to preserve existing protective immunity. However, depending on the approach used for directing expression of therapeutic proteins, preserving recipient immunity renders transferred gene-engineered HSPCs susceptible to immune attack [14] and failure of engraftment if they express neoantigens as a result of engineering. This is of relevance for both protein replacement therapies and approaches for instatement of immune tolerance, for example.

Previously we showed that one approach to limiting immune attack and preserving the integrity of transferred gene-engineered bone marrow (BM) and HSPCs was to restrict neoantigen expression to differentiated leukocytes, away from engrafting HSPCs [15]. Ubiquitous or ‘off-target’ expression of neoantigens in BM or HSC leads to their destruction in recipients with intact immunity [15, 16]. An alternative approach may be to limit the development of immune responses in HSPC recipients.

Here we continue to explore avenues to overcome immune resistance to engraftment of neoantigen-expressing gene-engineered HSPCs. Rapamycin is an immunosuppressant that functions by inhibiting mammalian target of rapamycin (mTOR) to block entry of T cells into the cell cycle which, unlike the calcineurin inhibitors cyclosporine and tacrolimus, does not inhibit TCR-induced Ca^2+^ signaling [17, 18], which is important for tolerance induction in some conditions [19, 20]. Both clinical and preclinical studies indicate that rapamycin is ‘tolerance-permissive’ in organ allograft and other settings, whereas cyclosporine and tacrolimus may inhibit the development of immune tolerance [21]. We report that a short course of rapamycin treatment is sufficient to prevent immune rejection of neoantigen-expressing BM and HSPCs in recipients with intact immunity. Protection of engrafting cells is mediated by suppression of transient effector differentiation in T cells undergoing peripheral tolerance induction elicited by HSPC-encoded antigen.

## Methods

### Mice

OT-I, 11c.OVA, MII.OVA and actin.OVA mice have been described elsewhere [7, 22–24]. Mice were maintained under specific pathogen-free conditions in the TRI Biological Resources Facilities, Brisbane, Australia. Nontransgenic C57BL/6 and B6.SJL-Ptprc^a^Pep3^b^/BoyJArc (B6.SJL) mice were purchased from ARC (Perth, Australia). Unless stated otherwise, in BM transplant experiments recipient mice were B6.SJL (CD45.1^+^) and donors were MII.OVA (CD45.2^+^), actin.OVA (CD45.2^+^) or nontransgenic (non-Tg) C57BL/6JArc (all CD45.2^+^) mice. To generate CD45.1^+^/CD45.2^+^ OT-I mice, B6.SJL mice were crossed with OT-I mice. All animal procedures were performed in accordance with the Australian Code for the Care and Use of Animals for Scientific Purposes and approved by the University of Queensland Animal Ethics Committee (projects DI/208/12; UQDI/296/14).

### Bone marrow and HPC transplantation

Donor mice were euthanised by CO_2_ narcosis and femurs and tibias collected into mouse tonicity (MT)-PBS. Bone marrow was flushed with MT-PBS/2.5% FCS, erythrocytes lysed (NH_4_Cl/TRIS buffer) and BM washed twice (MT-PBS/2.5% FCS). BM was resuspended in MT-PBS and injected i.v. (lateral tail vein) within 3 hours of total body irradiation (TBI; 300 cGy, ^137^Cs source). Unless stated otherwise, 10 × 10^6^ bulk BM was transferred. For high-dose irradiation experiments, the irradiation was delivered as two equal doses (550 cGy) 3 hours apart and mice were administered neomycin (1 mg/ml) in drinking water for 3 weeks. HSPCs were prepared by high-speed FACS sorting of lin^–ve^/c-kit^+ve^ cells to typically >95% purity from bulk BM. HSPC-depleted BM was lin^+ve^/c-kit^–ve^ cells prepared from BM by high-speed cell sorting.

### Immunosuppressant administration

Rapamycin (Rapamune, Wyeth Australia) was diluted in PBS and administered (0.6 mg/kg) by i.p. injection. Cyclosporine (Sandimmune, Novartis Pharmaceuticals Australia) was diluted in PBS and administered (25 mg/kg) daily by i.p. injection. Immunosuppressant administration commenced on the day of BM/HSPC transfer and continued daily for the following 21 days unless the experiment finished sooner. To determine the blood concentration of rapamycin, whole blood was collected in 0.5 M EDTA immediately prior to rapamycin administration on the days indicated and stored at −30 °C. LC-MS/MS analysis was performed using an Alliance HT LC system interfaced to a Quattro-Premier mass spectrometer (Waters Corporation, Milford, MA, USA).

### In-vitro and in-vivo assays

OVA/QuilA immunisation was as described previously [23]. Intracellular cytokine staining and in-vivo CTL assays were performed as described previously [7]. CFSE labeling was performed as described elsewhere [7] and proliferation indices calculated as described previously [25].

### Flow cytometry

Sample preparation for flow cytometry of BM, spleen and pooled lymph node (axillary, brachial, inguinal and mesenteric) was as described previously [7]. mAb were purchased from Biolegend, BD Biosciences and BioXcell (Lebanon, NH, USA) or were grown, purified and conjugated in-house. Analysis of peripheral blood for engraftment determination was performed using a bead-based counting assay as described previously [26]. Cytometric data were acquired using BD Canto or BD LSRII cytometers and analysed using Diva (BD) or Flow-Jo (Tree-Star) software.

### Statistical analysis

Student’s *t* test (two-tailed) was used for comparison of means and one-way ANOVA with Newman–Keuls or Tukey’s post test for multiple comparisons (GraphPad Prism 5 or Prism 6). *p* < 0.05 was considered significant.

## Results

### Increasing the dose of cells injected partially overcomes immune resistance to gene-modified BM

**Fig. 1:**
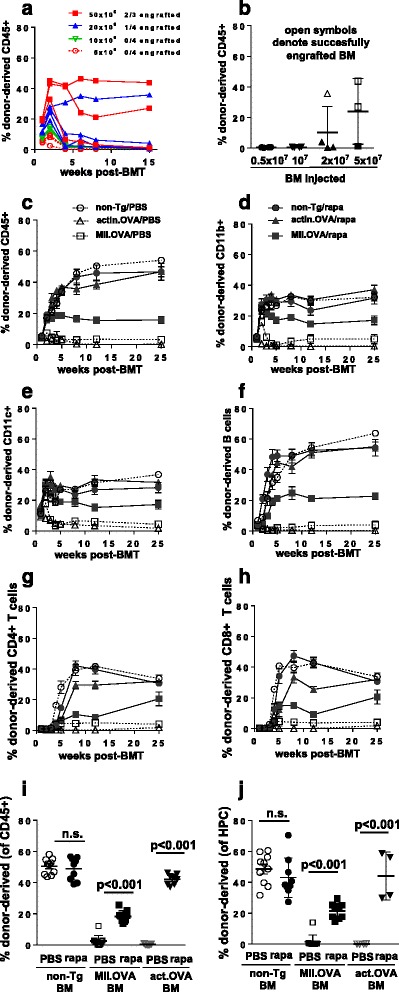
Rapamycin administration enables long-term multilineage engraftment of antigen-encoding BM under immune-preserving conditions. **a**, **b** Titrated doses of MII.OVA BM (5 × 10^6^, 10^7^, 2 × 10^7^ and 5 × 10^7^ cells) mice were transferred i.v. to B6.SJL mice under low-dose (300 cGy) irradiation. At designated time-points, engraftment was determined in peripheral blood leukocytes (PBL) by flow cytometry. **c**–**j** BM (10^7^ cells) from non-Tg, MII.OVA and actin.OVA mice was transferred i.v. to B6.SJL mice under low-dose irradiation (300 cGy TBI). Rapamycin or PBS was administered i.p. for 22 days commencing at BM transfer. Engraftment was determined for total leukocytes (**c**) or leukocyte subsets (**d**–**h**) within PBL at the indicated time-points and for total leukocytes in the spleen (**i**) and lin^–ve/^sca-1^+ve^/c-kit^+ve^ HSPC) in BM (**j**) 26 weeks after BMT. Data show individual mice or mean ± SEM of results from a single experiment (**a**, **b**), mean ± SEM of results pooled from three or four experiments (**c**–**h**) or individual mice with mean ± SEM pooled from three or four experiments (**i**, **j**). ANOVA with Tukey’s post test. *BM* bone marrow, *BMT* bone marrow transplant, *HPC* lin^–ve^/sca-1^+ve^/c-kit^+ve^ HSPC

Transfer of large doses of BM promotes engraftment of nonimmunogenic BM [27]. We tested whether increasing the dose of transferred immunogenic BM could overcome immune-mediated resistance to engraftment under conditions of low-dose irradiation where immune function is largely preserved [15]. Increasing the dose of BM transferred two-fold or five-fold over that normally transferred (10^7^ cells, approximately 10^6^ HSPCs/kg) led to a dose-dependent increase in donor-type leukocyte accumulation 2 weeks after BM transfer but this did not necessarily predict the final outcome with relation to long-term engraftment (Fig. 1a). Overall, however, increasing the dose of BM cells transferred increased the proportion of mice in which engraftment was successful (Fig. 1a, b), although engraftment was not observed in all recipients. Therefore, increasing the number of BM cells transferred, even to a ‘mega-dose’, did not reliably overcome immune resistance. While the trend suggested that further increases in BM dose may have led to engraftment in a higher proportion of recipients (Fig. 1a, b), this would be impractical for anything other than laboratory studies and unlikely to translate to a clinical scenario.

### Short-course rapamycin treatment permits engraftment of neoantigen-expressing BM under immune-retaining conditioning

**Fig. 2:**
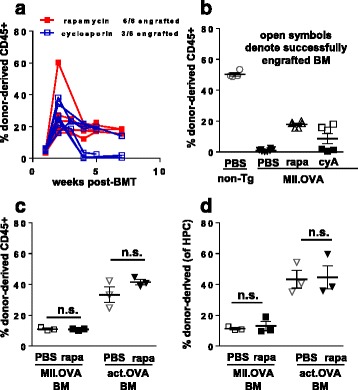
Rapamycin inhibits development of anti-graft immunity. **a**, **b** BM (10^7^ cells) from MII.OVA mice was transferred i.v. to B6.SJL mice under low-dose irradiation (300 cGy TBI). Rapamycin (*rapa*), cyclosporine (*CyA*) where indicated or PBS was administered i.p. for 22 days commencing at BM transfer as indicated and mice were studied in parallel. Engraftment was determined within PBL at the time-points indicated (**a**) and in the spleen 5 or 6 weeks after BMT (**b**). Data are individual mice (**a**) with mean ± SEM (**b**) pooled from two separate experiments. **c**, **d** Recipient mice (C57BL/6, CD45.2^+^) were irradiated (1100 cGy) and a 1:1 mixture of nontransgenic (B6.SJL; CD45.1^+^) and MII.OVA (CD45.2^+^) or nontransgenic (B6.SJL; CD45.1^+^) and actin.OVA (CD45.2^+^) mice transferred i.v. Mice were administered rapamycin (0.6 mg/kg) or PBS i.p. for 22 days commencing at BM transfer (*BMT*). Six weeks after BMT, relative accumulation of donor leukocytes in the spleen (**c**) and donor HSPCs in BM were determined (**d**). Data are from a single experiment representative of two performed where the effect of rapamycin was similar but overall engraftment differed. Data show individual mice with mean ± SEM. ANOVA with Tukey’s post test. *BM* bone marrow

We next tested alternative approaches to achieving effective engraftment of neoantigen-expressing, immunogenic gene-modified BM under low-dose irradiation. For this we chose a short, 3-week course of treatment with rapamycin or cyclosporine and compared engraftment of BM carrying transgenes encoding OVA expressed ubiquitously (actin.OVA) or in MHC class II+ cells (MII.OVA) where transient expression of MHC II, and consequently the OVA transgene, in HSC leads to failure of engraftment due to immune rejection. Addition of rapamycin promoted engraftment of both actin.OVA and MII.OVA BM, and donor-derived hematopoiesis was sustained for at least 6 months after transfer (Fig. 1c). Accumulation of donor-derived myeloid cells and DC was rapid and these populations accumulated to close to their final levels within 2–3 weeks of transfer (Fig. 1d, e). Donor-derived B-cell populations established more slowly but to higher levels overall, and T-cell populations stabilised slowly (Fig. 1f–h). Examination 26 weeks after transfer indicated accumulation of donor-derived leukocytes in peripheral blood leukocytes (PBL) reflected that lymphoid tissues (Fig. 1i) and examination of lin^–ve^,c-kit^+^ HSPC in BM (Fig. 1j) showed that accumulation of donor-derived leukocytes in the periphery reflected engraftment of donor-derived cells in the hematopoietic stem and progenitor cell compartment. In comparison with rapamycin, cyclosporine was much less effective and engraftment failed in 50% of MII.OVA BM recipients (Fig. 2a, b).

### Rapamycin promotes engraftment by limiting immune rejection

As shown in Fig. 1 controlling immune pressure by administration of rapamycin allows MII.OVA BM to engraft stably, but at a consistently reduced level compared with non-Tg or actin.OVA BM (Fig. 1c–j). This likely reflects reduced engraftment capacity of MII.OVA HSC [15] but could also potentially reflect transgene expression-induced endoplasmic reticulum (ER) stress [28] that might be relieved by rapamycin. Therefore, we tested the effect of rapamycin on the fitness of MII.OVA and actin.OVA BM in a competitive repopulation assay. Because recipient immunity is ablated by the lethal irradiation used, nonimmune effects of rapamycin are tested. When equal numbers of actin.OVA and non-Tg control or MII.OVA and non-Tg control BM were mixed and transferred to high-dose (1100 cGy) irradiated mice, MII.OVA BM showed a deficit compared with actin.OVA BM in leukocyte accumulation and engraftment in the HSPC compartment in PBS-treated controls (Fig. 2c, d) as expected (Fig. 1 [15]), consistent with reduced hematopoietic capacity. Notably, administration of rapamycin did not alter the relative pattern of donor-type leukocyte development in or between recipient groups (Fig. 2c, d), indicating that rapamycin did not provide a competitive advantage specific for MII.OVA engraftment and hematopoiesis in the absence of immune pressure.

### Antigen-expressing BM transfer paradoxically induces both BM rejection and transient T-cell unresponsiveness to BM-expressed antigen in the absence of rapamycin

**Fig. 3:**
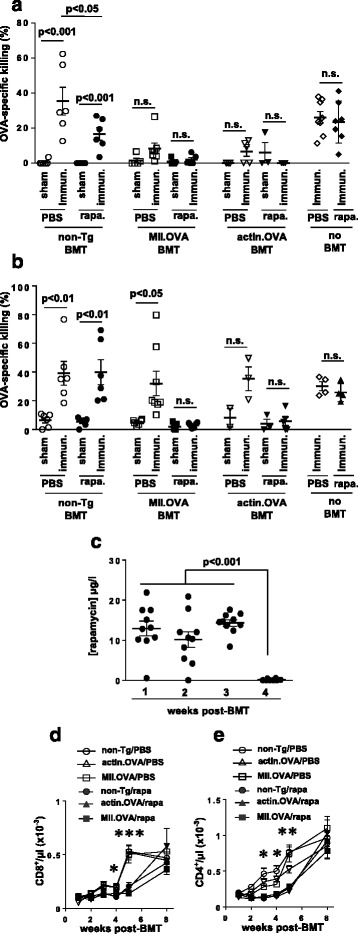
Stable engraftment is required for long-term tolerance. **a**–**e** BM (10^7^ cells) from non-Tg, MII.OVA and actin.OVA mice was transferred i.v. to B6.SJL mice under low-dose irradiation (300 cGy TBI). Rapamycin (*rapa*) or PBS was administered i.p. for 22 days commencing at BM transfer (*BMT*). Four weeks (**a**) or 25 weeks (**b**) after BMT, mice were sham (PBS/QuilA) or OVA (OVA/QuilA) immunised. Age-matched, unirradiated and untransplanted mice were immunised and analysed in parallel. One week later an in-vivo CTL assay was performed. Blood was collected at the indicated time-points and whole blood rapamycin concentration determined by HPLC (**c**). CD8^+^ (**d**) and CD4^+^ (**e**) T cells were enumerated in blood by flow cytometry. Data are pooled from three or four experiments and show individual mice with mean ± SEM (**a**, **b**), pooled from two experiments where mice from all experimental groups were pooled at each time-point and show individual mice with mean ± SEM (**c**) or pooled from three or four experiments and depict mean ± SEM (*n* = 8/group). ANOVA with Tukey’s post test. *PBS > rapa (*p* < 0.05), **PBS > rapa (*p* < 0.01 or greater), ***PBS > rapa (*p* < 0.001 or greater)

We reported previously that tolerance induction by antigen-encoding BM transfer requires establishment of stable engraftment. Therefore, we next tested whether enabling engraftment of antigen-expressing BM with a short course of rapamycin treatment promoted tolerance induction. We transferred BM using low-dose irradiation, where there is retention of immunity in recipients, with or without rapamycin administration. Four weeks after BM transfer, to determine antigen-responsiveness, mice that received BM were sham-immunised or immunised with OVA/QuilA and 1 week later an OVA-specific in-vivo CTL assay performed. CTL activity was compared with immunised but unirradiated, no BMT controls analysed in parallel. Recipients of non-Tg BM when immunised mounted significant CTL activity against OVA_257–264_-loaded targets which did not differ significantly from unirradiated, no BMT controls (Fig. 3a), indicating substantial immune responsiveness. Although rapamycin-treated non-Tg BM recipients generally showed induction of CTL activity in response to immunisation, this was reduced compared with their PBS-treated counterparts, indicating that rapamycin administration somewhat inhibited the development of CTL activity in BM recipients. Interestingly, rapamycin inhibition of CTL induction relative to PBS controls occurred only in irradiated mice and not in unirradiated, no BMT controls (Fig. 3a). Recipients of MII.OVA or actin.OVA BM, when immunised, developed little OVA-specific CTL activity regardless of whether they had been administered rapamycin or not. This was surprising because, in the absence of rapamycin, MII.OVA and actin.OVA BM fails to engraft, suggesting that in the absence of rapamycin treatment there is induction of anti-graft immunity, but unresponsiveness also develops. Inhibition of CTL induction in rapamycin-treated MII.OVA and actin.OVA BM recipients is consistent with induction of tolerance in the presence of successful antigen-encoding BM engraftment [15] but could potentially also reflect a compound effect of OVA-encoding BM engraftment and rapamycin treatment. In contrast, the failure of CTL induction in recipients of OVA-encoding/expressing BM that were not administered rapamycin could reflect several effects.

### Stable long-term tolerance to BM-expressed antigen requires rapamycin-facilitated engraftment

Before investigating the mechanisms that might underlie failure of CTL induction 4 weeks after BM transfer, we wished to determine firstly whether unresponsiveness was transient, indicating a peri-BMT effect, or whether unresponsiveness to OVA was long-lasting irrespective of rapamycin administration. To achieve this we performed BM transfers, but waited 25 weeks before mice were sham-immunised or immunised with OVA/QuilA and then OVA-specific in-vivo CTL activity was tested 1 week later. In this setting, in recipients of non-Tg BM regardless of rapamycin treatment there was strong CTL activity, equivalent to that in no BMT controls, induced by immunisation. In MII.OVA BM recipients, immunisation elicited CTL activity only in PBS-treated but not rapamycin-treated recipients (Fig. 3b). A similar trend was observed in the small number of actin.OVA BM recipients analysed (Fig. 3b); however, this was not significant in the PBS-treated group due to the low number of mice tested. In contrast, in rapamycin-treated recipients of MII.OVA and actin.OVA BM, CTL induction by immunisation was almost completely damped (Fig. 3b). This demonstrates that although there was modulation of OVA responsiveness soon after BMT in recipients of OVA-encoding BM regardless of rapamycin treatment, stable, long-lasting T-cell tolerance only occurred when OVA-expressing BMT was combined with rapamycin to facilitate stable engraftment of OVA-expressing BM.

### Rapamycin treatment delays T-cell recovery after BMT

To investigate why OVA responsiveness may have been modulated soon after BMT in rapamycin-treated non-Tg BM recipients and perhaps in OVA-encoding BM recipients, we first determined whether residual rapamycin may play a role. The rapamycin concentration in whole blood was within the clinical therapeutic range (6–15 μg/l) in most mice throughout the treatment period but had diminished to undetectable levels 7 days after cessation of treatment (Fig. 3c) when mice in some experiments mice were immunised. Examination of the T-cell repopulation kinetics after irradiation showed that rapamycin administration delayed recovery of CD8^+^ and CD4^+^ T-cell populations from the partial lymphopenia induced by low-dose irradiation (Fig. 3d, e). This was most prominent during or soon after cessation of rapamycin treatment (Fig. 3d, e). By 5 weeks after rapamycin cessation, T-cell recovery was approximately equal in all groups. This suggests that rapamycin-treated recipients of non-Tg BM exhibit impaired antigen-responsiveness due to reduced T-cell repopulation. However, this reflects total CD8^+^ or CD4^+^ T-cell number and does not necessarily indicate relative repopulation with OVA-specific T cells or OVA responsiveness specifically, nor explain why PBS-treated recipients of OVA-encoding/expressing BM exhibit CTL unresponsiveness 4–5 weeks after BMT. When the presence of CD4^+^CD25^+^FoxP3^+^ regulatory T cells (Treg) was analysed, we found no evidence that Treg were preferentially expanded in any group, suggesting that Treg were not responsible for the unresponsiveness observed.

### Development of stable long-term tolerance engraft is associated with the extended presence of cognate antigen

**Fig. 4:**
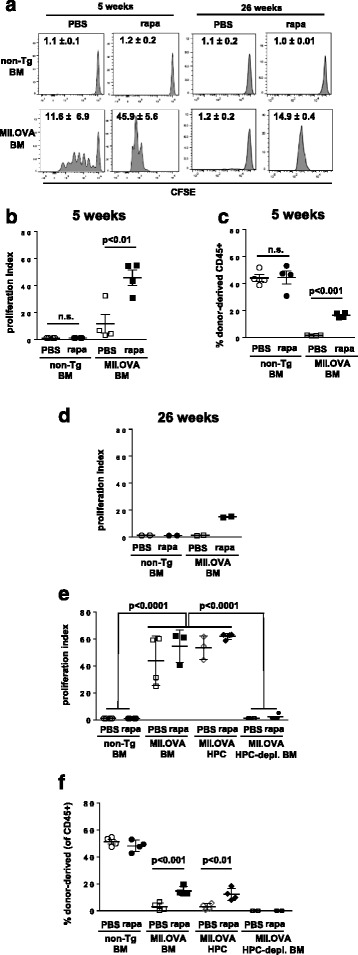
Presence of antigen after transfer of MII.OVA bone marrow induces transient antigen specific tolerance. **a**–**c** BM (10^7^ cells) from non-Tg and MII.OVA mice was transferred i.v. to B6.SJL mice under low-dose irradiation (300 cGy TBI). Rapamycin (*rapa*; 0.6 mg/kg) or PBS was administered i.p. for 22 days commencing at BMT. Five or 26 weeks after BMT, CFSE-labeled OT-I T cells (5 × 10^6^) cells were transferred i.v. and 3 days later CFSE dilution determined by flow cytometry of lymph node cells. Data show representative histograms and proliferation index (mean ± SEM) for OT-I in pooled lymph nodes from each group (**a**) or individual mice at 5 weeks where *bars* denote mean ± SEM (**b**) and the level of engraftment in the spleen for individual mice where *bars* denote mean ± SEM (**c**) or at 26 weeks (**d**). Pooled from two experiments with 2 mice per group (5 weeks) or from a single experiment with 2 mice per group (26 weeks). **e**, **f** Whole BM, HPC (Lin^–ve^,c-kit^+^; 2 × 10^5^ cells) and HPC-depleted BM (Lin^+ve^,c-kit^–^; 10^7^ cells from non-transgenic or MII.OVA mice was transferred to B6.SJL (CD45.1^+^) mice under low-dose irradiation. Four weeks after BMT, CFSE-labeled OT-I T cells (5 × 10^6^) were transferred i.v. and 3 days later CFSE dilution determined by flow cytometry. Data show proliferation index of OT-I cells (**e**) and engraftment levels in the spleens of individual mice (**f**) pooled from two experiments. *Bars* denote mean ± SEM. ANOVA with Tukey’s post test. *BM* bone marrow, *HPC* defines lin^–ve^/c-kit^+ve^ hematopoietic progenitor cells

It has been proposed that maintenance of T-cell tolerance requires stable engraftment of transferred HSPCs in order that a long-term source of ongoing tolerogenic antigen is generated [15]. We sought to determine whether this might underlie the effectiveness of transient rapamycin administration. To probe for the presence of OVA, CFSE-labeled OT-I T cells were transferred 5 weeks after transfer of non-Tg or MII.OVA BM with or without rapamycin treatment and CFSE dilution determined 3 days later. In recipients of MII.OVA BM, CFSE dilution in OT-I T cells (Fig. 4a, b) indicated that OVA-specific T cells recognised their cognate antigen regardless of whether recipients had been treated with rapamycin or not. However, the extent of cell division was substantially greater and more uniform in MII.OVA BM recipients if treated with rapamycin (Fig. 4a, b), consistent with the higher levels of donor-type leukocyte development relative to their PBS-treated counterparts (Fig. 4c). When similar assays were performed 26 weeks after BMT, OT-I division indicated the presence of immunologically relevant OVA in MII.OVA BM recipients that had been transiently administered rapamycin at the time of BM transfer, but not in the PBS-treated counterparts (Fig. 4d), in keeping with the stable engraftment observed in rapamycin-treated recipients (Fig. 1). This indicated that immunologically relevant OVA was present only transiently in PBS-treated MII.OVA BM recipients.

### OVA arises from transferred HSPCs in MII.OVA BM recipients

Paradoxically, in the absence of rapamycin, transfer of OVA-expressing BM induces not only transient tolerance but also promotes the ultimate rejection of engrafting HSPCs. It is possible that this unresponsiveness is due to transient expression of OVA in PBS-treated recipients of OVA-expressing BM because immunologically active OVA is not present 26 weeks after BMT (Fig. 4a, d). Because cells within the transferred whole BM prepared from MII.OVA donors express OVA [15] and could potentially induce tolerance or immunity [29], we next determined whether the immunologically relevant OVA present in PBS-treated MII.OVA BM recipients that might have contributed to induction of rejection and the development of unresponsiveness to OVA was derived from the BM graft, HSPCs within the graft or from the non-HSPC component of the graft. Whole BM, HSPCs or HSPC-depleted BM from MII.OVA and BM from non-Tg mice was transferred to low-dose irradiated recipients with or without rapamycin treatment and 4 weeks after BMT CFSE-labeled OT-I T cells were transferred to detect the presence of immunologically relevant OVA. OT-I proliferated only in recipients of MII.OVA whole BM or HSPCs and not HSPC-depleted BM (Fig. 4d). This suggests the source of OVA in MII.OVA BM recipients was HSPCs, most likely through engraftment and/or development of progeny (Fig. 1). Based on the reduced proliferation of OT-I T cells at week 5 (Fig. 4b) relative to week 4 (Fig. 4e), residual OVA is cleared quickly after engraftment failure in PBS-treated recipients.

### Rapamycin inhibits development of transient effector function during tolerance induction

**Fig. 5:**
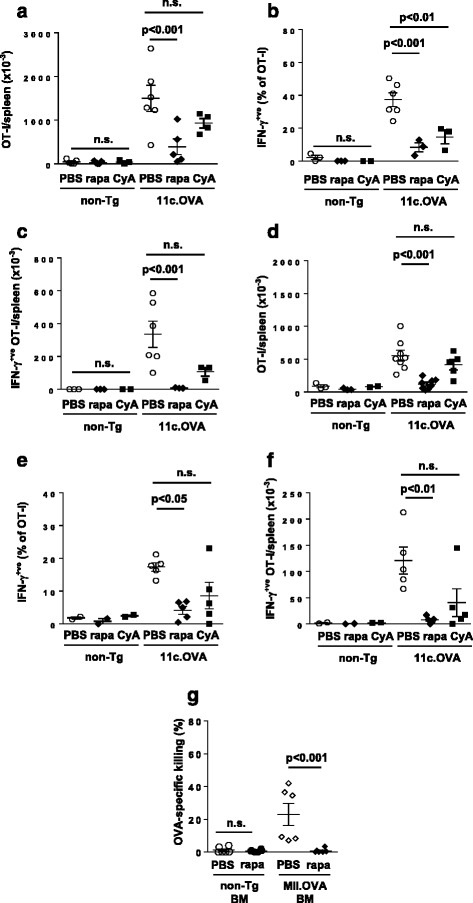
Rapamycin inhibits T-cell effector differentiation during tolerance induction. **a**–**g** Naive OT-I cells were transferred to 11c.OVA mice and rapamycin (*rapa*), cyclosporine (*CyA*) or PBS was administered 3 hrs before adoptive transfer and daily thereafter. Three days (**a**–**c**) or 7 days (**d**–**f**) after OT-I transfer, spleens were harvested and OT-I cells enumerated in the spleen (**a**, **d**), the proportion of OT-I T cells producing IFN-γ was determined by intracellular cytokine staining (**b**, **e**) and the total number of IFN-γ-producing OT-I T cells per spleen calculated (**c**, **f**) were determined. **g ** BM (10^7^ cells) from non-Tg and MII.OVA mice was transferred i.v. to B6.SJL mice under low-dose irradiation (300 cGy TBI). Rapamycin (0.6 mg/kg) or PBS was administered i.p. for 16 days commencing at BM transfer. The day after rapamycin cessation an in-vivo CTL assay was performed. Data represent individual mice pooled from two experiments (mean ± SEM). ANOVA with Tukey’s post test. *BM* bone marrow

In vivo, T cells fated for peripheral tolerance induction undergo a period of abortive proliferation followed by a period of population contraction during which most antigen-specific T cells are deleted. Significantly, during the expansion phase T cells transiently exert some degree of effector function [23, 30, 31]. Therefore, we explored the effects of rapamycin using a well-defined mouse model of tolerance in which OVA is expressed tolerogenically by DC (11c.OVA mice) and where the development of transient effector function by CD8^+^ T cells undergoing is well characterised [7, 23]. In this setting, 3 days after transfer into the tolerogenic 11c.OVA environment, OVA-specific CD8^+^ OT-I T cells had proliferated and expanded substantially in control PBS-treated mice compared with non-Tg controls (Fig. 5a). Furthermore, a large proportion of the OT-I population in the tolerogenic 11c.OVA environment produced IFN-γ as an indicator of transient effector differentiation (Fig. 5b). Administration of rapamycin significantly reduced expansion of OT-I T cells in 11c.OVA recipients (Fig. 5a) and inhibited effector differentiation indicated by reduced acquisition of IFN-γ production (Fig. 5b) when analysed 3 days after OT-I transfer. Rapamycin administration strikingly reduced the total number of IFN-γ^+ve^ (effector-differentiated) OT-I T cells present in the 11c.OVA recipients 3 days after transfer (Fig. 5c). In contrast, administration of cyclosporine in this setting limited IFN-γ production but had weaker effects on expansion of OT-I T cells such that the total number of IFN-γ^+ve^ (effector-differentiated) OT-I T cells was not significantly reduced. This difference between rapamycin and cyclosporine was maintained at least to 7 days after transfer (Fig 5d–f).

To identify whether the action of rapamycin was to inhibit differentiation of effector function in the ‘intrinsically tolerogenic’ MII.OVA BM transfer setting, non-Tg or MII.OVA BM was transferred to low-dose (300 cGy) irradiated recipients with or without rapamycin administration, and spontaneous CTL activity that had developed 17 days later, when BM rejection has commenced (Fig. 1, Additional file 1) and engraftment is failing (Fig. 4c), was tested. No CTL activity was detected in recipients of non-Tg BM as expected (Fig. 5g). In contrast, in PBS-treated MII.OVA BM recipients, substantial killing of OVA_257–264_-pulsed targets was observed, which was not present in rapamycin-treated MII.OVA BM recipients (Fig. 5g).

Rapamycin therefore acts to prevent the emergence of the transient effector function elicited in OVA-specific T cells as a component of tolerance induction. Ironically, engraftment appears to proceed initially giving rise to donor-derived antigen expressing DC (Additional file 1) and while these are potentially tolerogenic, the transient effector function elicited in the early phase of tolerance induction leads to rejection of antigen-expressing HSC. In the absence of transient immune suppression to control this, engraftment fails and induction of long-term tolerance that requires ongoing antigen expression by the progeny of successfully engrafted OVA-encoding HSPCs stalls before fully developing.

## Discussion

Increasing the clinical applicability of HSPC-based gene therapy is an important goal that will maximise the usefulness of this potentially powerful therapeutic. Defining approaches that reduce the toxicity of HSPC transfer-associated procedures is a key requirement. Enabling high levels of engineered HSPC engraftment and subsequent leukocyte development through either increasing the competitive advantage of transferred engineered HSPCs or opening ‘engraftment niches’ in the recipient whilst reducing treatment toxicity is one important focus. However, here we have focused on overcoming the challenges of immune resistance that is a consequence of attempts to achieve the desired outcome of preserving recipient immune function during HSPC-mediated gene transfer. Here we show that HSPC-based approaches capable of inducing immune tolerance which could, for instance, alleviate autoimmune diseases or allergies can be hindered by the development of transient effector function in the very T cells that are targeted for inactivation by the procedure. Development of transient effector function is a normal component of the early phase of tolerance induction in T cells [23, 30, 31] but, using rapamycin, we show this can be readily controlled by a short course of appropriate immunosuppressant administration. Limiting effector differentiation during the critical peritransfer period facilitates engraftment and leads to establishment of long-term tolerance that does not require additional immunosuppression for maintenance.

A notable observation was that rapamycin was highly effective at promoting engraftment of BM expressing a neoantigen under the immune-preserving conditions used, but that cyclosporine was much less effective. This is supported by similar results in an allogeneic BM transplant setting [32]. Competitive repopulation assays ruled out that rapamycin provided a nonimmunological engraftment-enhancing effect to transgene-encoding HSPCs. While rapamycin did not appear to act on HSPCs, agents that protect the HSPC niche from radiation-induced damage or foster hematopoiesis or myelopoiesis/erythropoiesis, such as lysophophatidic acid [33, 34], might promote post-HSPC transfer engraftment. Rapamycin or its analog everolimus has been reported as ‘tolerance-permissive’ in organ allograft [21] and other settings [35, 36], and it is possible that this underlies these observations. Effectiveness here, however, appeared to be associated with the capacity of rapamycin to inhibit both expansion and transient effector function elicited by tolerogenic antigen presentation. Cyclosporine, by contrast, poorly controlled expansion of T cells undergoing tolerance induction. In a small number of cyclosporine-treated animals tested, responsiveness to OVA was inversely correlated with the level of MII.OVA BM engraftment present. This is in line with previous conclusions that, under conditions where potentially tolerogenic BM is transferred, tolerance is related to successful engraftment [15] rather than perhaps the immunosuppressant used. It might be that the effectiveness of rapamycin as an anti-proliferative agent for T cells [37] is the critical factor, particularly here where tolerogenic rather than immunogenic antigen presentation is present. The anti-proliferative effects of rapamycin impaired T-cell recovery after irradiation and BM transfer, and this could potentially also contribute. Interestingly, the extent of the rapamycin-induced delay in T-cell reconstitution differed somewhat between CD8^+^ and CD4^+^ T cells. Why CD4^+^ T cells appear to be more affected remains unclear. However, a possible explanation is that the homeostatic proliferation which contributes to T-cell recovery after low-dose irradiation [38] is modulated by the differential sensitivity of distinct homeostatic cytokines such as IL-7 and IL-15 to rapamycin-mediated inhibition of mTOR between different T-cell subsets [39–41].

Moderate doses of irradiation can lead to BM transfer-associated regulatory T cell (Treg) expansion [42] which could potentially be enhanced by rapamycin. While not shown, we found no evidence that Treg expansion contributed to rapamycin-mediated effects. However, in other studies the irradiation dose required for expansion of antigen-specific CD4^+^CD25^+^FoxP3^+^ Treg was higher (>450 cGy) [42] or myeloablative doses of irradiation were used for CD8^+^Foxp3^+^ Treg induction/expansion [43], and the latter study used an allogeneic transplant setting and alloantigen was required for Treg generation/expansion. In other studies exploring transfer of antigen-encoding BM, no evidence of Treg induction has been reported [10] unless CD4^+^ TCR transgenic T cells are included [44, 45]. Administration of rapamycin has also been shown to induce or expand Treg in vivo, but in many cases this has been in the presence of coadministered antigen and/or a source of exogenous IL-2 [35, 36] and strong inflammatory signals may promote this effect [46]. The rapamycin treatment period of 3 weeks chosen here was based on our previous studies showing that induction of peripheral CD8^+^ T-cell tolerance is complete within 2–3 weeks of antigen encounter [7] and, although not tested here, it is possible a shorter course is also effective.

In the absence of rapamycin, engraftment and leukocyte development is transient, proceeds for approximately 2 weeks, but ultimately fails (Fig. 1) due to immune rejection. Paradoxically, despite immune rejection of OVA-encoding BM, OVA-specific CD8^+^ T cells are either deleted or rendered antigen-unresponsive as recipients fail to develop CTL activity in response to immunisation for some time after BM rejection. We conclude this is mediated by a transient presence of HSPC-derived OVA manifesting in the absence of rapamycin. However, once OVA is no longer present, immune responsiveness recovers likely through thymic export of OVA-specific T cells which is prevented by central tolerance in the presence of stable OVA-encoding BM engraftment. Whether HSPCs directly, or their progeny or host APC, are responsible is yet to be defined. It is also possible in this setting that the CTL activity elicited against transferred HSPCs is integral to tolerance induction by inducing apoptosis-mediated release of tolerogenic antigen as reported for CTL attack of pancreatic islet β cells [47].

The transient effector state that occurs in T cells early during tolerance induction [23, 30, 31] likely reflects a partially differentiated state that occurs during peripheral tolerance induction while T cells are integrating environmental signals and the final cell is being determined. The presence of transient T-cell effector function during ‘tolerisation’ is likely of little consequence under normal steady-state conditions because only a small number of potentially pathogenic autoreactive T cells would be undergoing tolerance induction at any one time and the number of target cells would be numerically much larger. Although immune preserving, the conditioning used in the BM transfer setting tested here results in partial lymphopenia which has the potential to promote the deleterious effects of the transient effector function elicited [48], and this may be particularly evident when target cells, in this case engrafting HSPCs, are present in low numbers. Under these circumstances, controlling transient effector function appears critical and rapamycin may be particularly effective through the combined effects on proliferation and effector differentiation discussed.

Our previous studies and those of others indicate that long-lasting expression of BM-encoded antigen is crucial to maintain tolerance [15, 49, 50]. Our data are consistent with a conclusion that many cellular sources of antigen are tolerogenic, but a critical window exists where the cellular antigen sources require protection from transient effector T-cell attack to establish tolerance. Supporting this there is emerging evidence in humans that a persistent source of antigen maintains BM-induced tolerance, although the source of antigen may not need to be BM-derived cells [51]. Lessons learned here that transient immunosuppression, using appropriate tolerance-permissive agents, provides a window of opportunity for tolerance induction may be applicable to a range of gene-therapy settings where immunity is preserved and the potential for immune resistance to therapeutic proteins is generated. Potential settings include limiting immune responses to therapeutically expressed proteins, facilitating viral vector-mediated gene transfer where the viral vector may be immunogenic approaches or preventing immune responses to the products of genes edited using, for example, CRISPR/Cas9 technologies.

## Conclusions

A short course of rapamycin promotes the engraftment of gene-engineered, antigen-expressing BM by suppressing the acquisition of transient T-cell effector function during peripheral tolerance induction that is elicited by HSPC-encoded antigen. By facilitating engraftment, short-course rapamycin permits development of long-term stable engraftment which maintains T-cell tolerance through a combination of central and peripheral mechanisms. Such short-course treatment with conventional immunosuppression represents a clinically applicable approach to overcoming immune resistance to genetically engineered bone marrow when immune-preserving conditions are employed.

## Additional file

Additional file 1: is **Figure S1.** showing additional analysis of data provided in the main body of the manuscript. (PDF 573 kb)

## Abbreviations

APC: Antigen-presenting cells
BM: Bone marrow
BMT: Bone marrow transplant
CFSE: Carboxyfluorescein succinimidyl ester
CTL: Cytotoxic T lymphocyte
HSPC: Hematopoietic stem and progenitor cell
Non-Tg: Nontransgenic
TCR: T cell receptor
Tg: Transgenic
