# Supplementary material for: Short-course rapamycin treatment enables engraftment of immunogenic gene-engineered bone marrow under low-dose irradiation to permit long-term immunological tolerance
Source: Stem Cell Res Ther. 2017 Mar 9;8:57. doi: 10.1186/s13287-017-0508-3 (PMC5345164; doi:10.1186/s13287-017-0508-3)
Supplement: Additional file 1: — is Figure S1. showing additional analysis of data provided in the main body of the manuscript. (PDF 573 kb) [file 13287_2017_508_MOESM1_ESM.pdf]

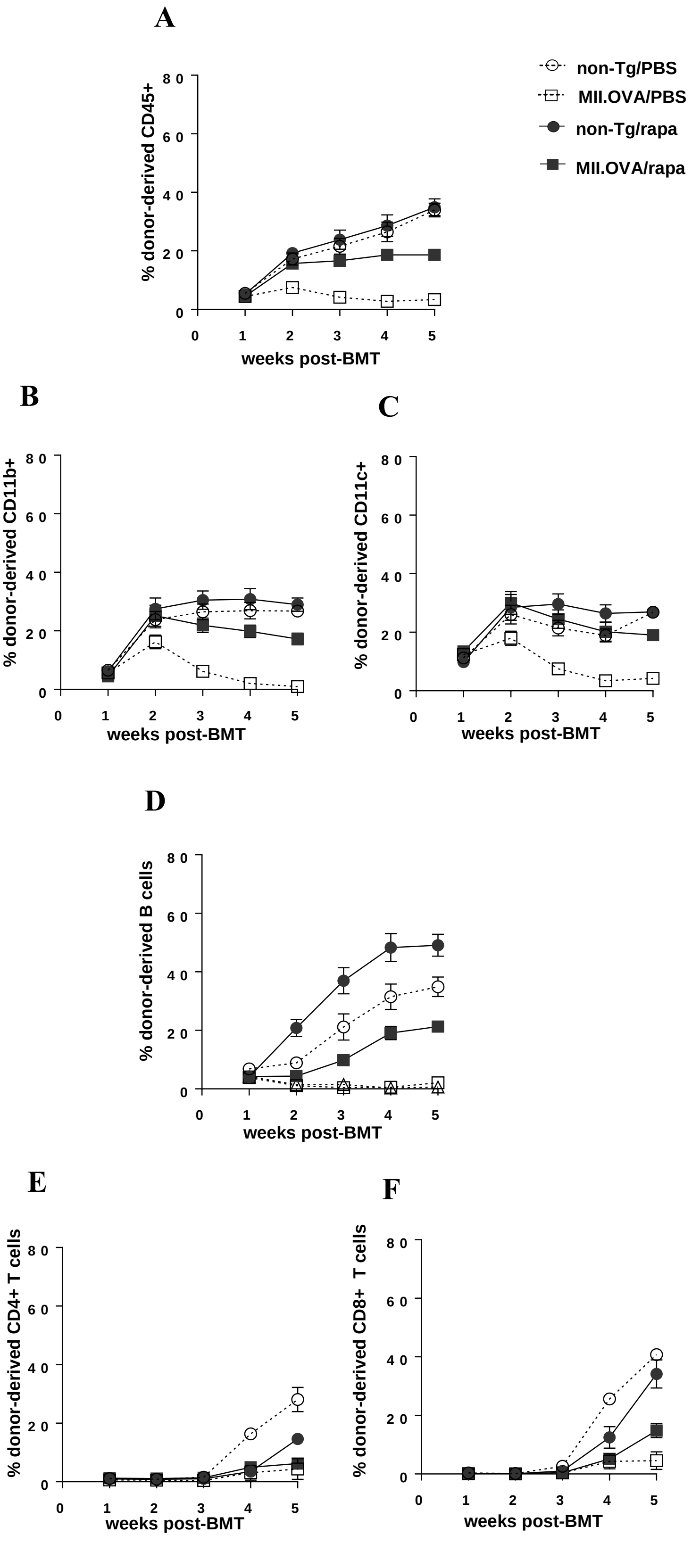


**Figure S1.**

Data are as presented in Figure 1 but with the first 5 weeks shown. BM ($10^7$) from non-Tg and MII.OVA mice was transferred i.v. to B6.SJL mice under low-dose irradiation (300cGy TBI). Rapamycin or PBS was administered i.p. for 22 days commencing at BM transfer. Engraftment was determined for total leukocytes (**A**) or leukocyte subsets (**B-F**) within PBL at the indicated timepoints. Data show mean ± SEM of results pooled from 3-4 experiments
